# Acute surgical‐like abdomen as a gastrointestinal manifestation of COVID-19 infection: a case report in Colombia

**DOI:** 10.1186/s12876-021-01762-0

**Published:** 2021-04-22

**Authors:** Fernando Sierra-Arango, Cathalina Vergara-Cabra, Mariana Vásquez-Roldan, Erika D. Pérez-Riveros

**Affiliations:** 1 Clinical Studies and Clinical Epidemiology Division, Digestive Endoscopy Division, Fundación Santa Fe de Bogotá, Bogotá, Colombia; 2 Gastroenterology and Hepatology Department, Fundación Santa Fe de Bogotá, Bogotá, Colombia; 3 Gastroenterology and Hepatology Department and Clinical Studies and Clinical Epidemiology Division, Fundación Santa Fe de Bogotá, Bogotá, Colombia; 4 Fundación Santa Fe de Bogotá, Calle 119a# 7-49, Bogotá, Colombia

**Keywords:** SARS-CoV-2, Acute abdomen, Surgical Procedure, Abdominal pain

## Abstract

**Background:**

The SARS-CoV-2 pandemic is a considerable public health problem, which has caused a burden on health systems in many countries. Despite the existence of multiple studies on the different digestive symptoms and their relationship with this disease, it is still vital to highlight the severity of the different symptoms, the need to diagnose it properly and quickly. Currently in Colombia there are no writings that highlight the above.

**Case presentation:**

This article reports the case of a 37-year-old female patient, with no important history, who consulted for 10 h of a generalized intense abdominal pain, of sudden onset, associated with multiple stools of diarrheal consistency, and no respiratory symptoms and no epidemiological exposure. Physical examination with intense pain in the colic frame with tenderness. It was decided to rule out surgical pathology and a CT scan was performed finding no evidence of acute intra-abdominal pathology, but with a peripheral alveolar, and ground-glass opacities at lung bases, classic COVID-19 radiological pattern, confirmed by a positive RT-PCR for SARS-CoV-2, leading to consider that the gastrointestinal symptoms were secondary to this infection. Symptomatic management was given with subsequent improvement.

**Conclusions:**

It is extremely important to present this first case report of a young female COVID-19 patient with an acute abdominal pain as the main clinical manifestation, that almost culminates in a surgical procedure; demonstrating the scope of gastrointestinal symptoms secondary to SARS-CoV-2 infection.

## Background

The SARS-CoV-2 pandemic has exponentially increased to more than 121,209,510 confirmed cases worldwide, including 2,680,469 deaths reported to the WHO [1, 2]. Increasing the need to acquire knowledge through research and demanding a rapid response from health systems and medical personnel.

COVID-19 generates a wide spectrum of symptoms, from completely asymptomatic patients to severe manifestations mainly pulmonary such as pneumonia, respiratory failure, shock, or dysfunction of the multi-organ system. Gastrointestinal (GI) symptoms like diarrhea, nausea and vomiting are also common, even a recent study reported that patients who presented with digestive manifestations had a worse prognosis [3, 4].

The pathophysiology of gastrointestinal manifestations remains unclear. Evidence suggests that the gastrointestinal tract could be a viral target for SARS-CoV-2 infection because this pathogen infects host cells through the angiotensin-converting enzyme 2 (ACE2) receptor that is expressed in various organs, including the lungs, heart, kidneys, and intestine. Most of these receptors are found on the epithelial cells of the esophagus and on the absorbent enterocytes of the ileum and colon. Once the virus enters the cell, it has the potential to generate a release of cytokines and chemokines, which triggers an acute intestinal inflammation characterized by infiltration of neutrophils, macrophages and T cells. This would explain the microvascular dysfunction associated with tissue edema and sometimes with pro-coagulant state or bleeding which could explain the clinical presentation of some patients [3, 5].

Below we present the clinical case of a young patient with acute abdominal pain, which almost culminated in a surgical procedure and after an adequate medical approach, she was diagnosed with COVID-19 and given the appropriate management. The foregoing demonstrates the extent of gastrointestinal symptoms secondary to SARS-CoV-2 infection and clarifies the different initial symptoms of this disease for patients, allowing not only to quickly identify and isolate these patients, but also to better understand the disease and make management approaches more consistent with the clinic and pathophysiology of the disease [5, 6].

## Case presentation

A 37-year-old female patient, with no significant history, who consulted for 10 h for severe generalized abdominal pain of sudden onset, associated with multiple diarrheal stools without mucus or blood (6 stools in the last hour), without fever, nausea or vomiting. She had no respiratory symptoms or epidemiological exposure at that time. Upon admission to the physical examination, the patient was stable, moderately dehydrated, and with mild tachycardia. During abdominal palpation, she had only severe crampy pain with tenderness, apparently no peritoneal irritation.

The laboratory results included a slightly normal blood count, normal arterial gases, negative pregnancy test, normal urinalysis and liver profile with mild elevation of transaminase (Table 1). Analgesic management with opioid and antispasmodics were initiated given the intensity of pain. An endoscopy was performed showing chronic gastritis and biopsies confirming this condition so she was discharged with ambulatory management.

**Table 1:** Paraclinics report

	Results	Reference values
Paraclinic		
Amylase	67,00 U/L	29,00- 103,00
Aspartate aminotransferase	58,00 U/L	0,00–35,00
Alanine aminotransferase	72,00 U/L	0,00–35,00
Total bilirubin	0,34 mg/dl	0,30 − 1,00
Direct bilirubin	0,06 mg/dl	0,00–0,20
Leukocytes	5,80 10^3/ul	5,00–10,00
Neutrophils	4,30 10^3/ul	1,40–6,50
Lymphocytes	0,80 10^3/ul	1,20–3,40
Hemoglobin	14,10 g/dl 10^3/ul	12,00–16,00
Hematocrit	43,30 % 10^3/ul	36,00–48,00
Platelets	191,00 10^3/ul	150,00–450,00
Human chorionic gonadotropin	0,48 mIU/ml	0–0,6
Arterial gases		
PH	7,51	7,40–7,46
PCO2	31	28,00–32,00
PO2	71	60,00–100,00
HCO3	24,7	--
FIO2	21	--
Lactate	1,1	--
Base excess	1,7	--
SPO2	96 %	90–100 %
PAFI	338	--

Paraclinics that were made to the patient with reference values, in international units

**Fig. 1:**
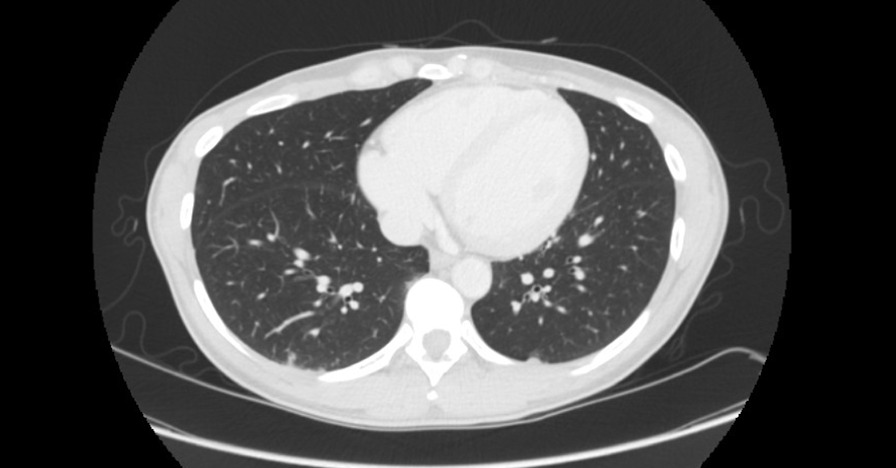
Contrasted abdomen CT scan: A peripheral alveolar, and ground-glass opacities at lung window

**Fig. 2:**
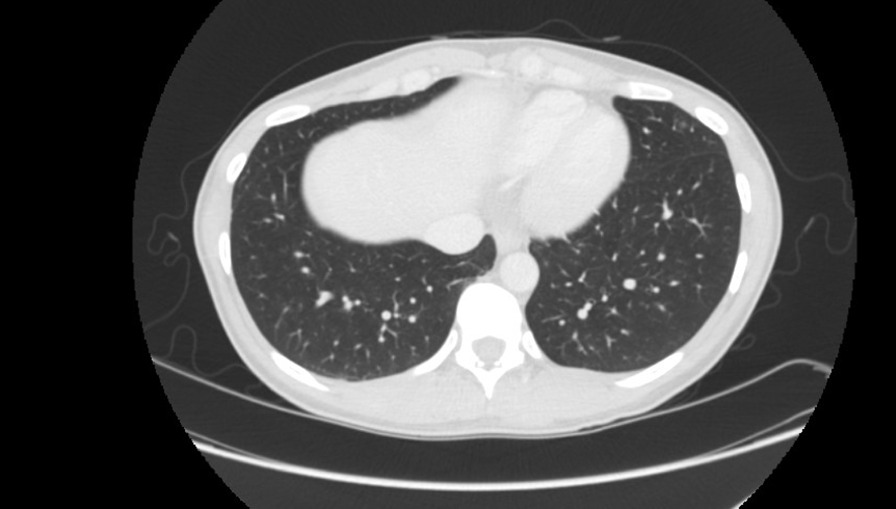
Contrasted abdomen CT scan: A peripheral alveolar, and ground-glass opacities at lung window

The patient has to consulted again to the emergency room due to the persistence of the symptoms. Therefore, it was decided to rule out surgical pathology and physicians performed a contrasted abdomen CT scan. The report showed no imaging evidence suggesting acute intra-abdominal pathology, but with a peripheral alveolar, and ground-glass opacities at lung bases (Figs. 1 and 2), classic COVID-19 radiological pattern, one week ago a RT-PCR SARS-CoV-2 test was performed and the result was positive.

The patient was diagnosed with COVID-19 because of the images and lab test leading us to consider that the gastrointestinal symptoms were secondary to this infection. Symptomatic management was given with subsequent improvement so it was decided to discharge the patient after two days, with recommendations.

## Discussion and conclusion

Throughout this pandemic, emphasis has been placed on respiratory symptoms, having the highest prevalence, however, other clinical manifestations should be considered, including gastrointestinal symptoms [6, 7]. The epithelial cells of the stomach and liver cells express angiotensin converting enzyme 2 (ACE2), the main receptor for SARS-CoV-2 through which it infects humans and through which it induces apoptosis and pyroptosis. Leading to cell damage and dysfunction of the microcirculation resulting in cases of bleeding and inflammation in the intestine that explain the symptoms of pain [8].

Therefore, it is important to highlight two aspects based on the present case. First the needing of a proper diagnosis because some patients can simulate an acute abdomen and be inappropriately taken for surgery, and second the early identification of symptoms related to COVID-19 to avoid contagion. The characteristics of gastrointestinal symptoms are more insidious than respiratory, making them easy to miss. Some patients may have only gastrointestinal manifestations, which makes it confusing for patients who may ignore it and not consult it, and for care personnel who do not suspect the true origin of the disease. Some of the patients even continue to excrete the virus in the stool with negative respiratory samples, further complicating the diagnostic approach and leading to potential problems for themselves and individuals with whom they came into contact becoming them as the major drivers of the pandemic emergency [3, 9].

Thus, patients diagnosed with COVID-19 are more susceptible to developing micro-thrombosis and even ischemia of the vascular bed of the gastrointestinal tract [9, 10] due to the coagulopathy process that is triggered [11–13]. In our case, the patient did not present respiratory symptoms and the approach was made based on abdominal CT imaging findings in which alveolar opacities were observed in ground glass patterns in the lung bases. Given the above, gastrointestinal infection by COVID-19 should be considered in all patients who come to the emergency room [6, 7, 14]. Multiple studies recommend that the SARS-CoV-2 test be considered a protocol for patients presenting with gastrointestinal symptoms, especially severe acute abdominal pain that suggests surgical pathology as a differential diagnosis. In this way, complications of both the pathology itself and the diagnostic process can be avoided and an epidemiological fence can be made in time.

## Data Availability

Data sharing is not applicable to this article as no datasets were generated or analyzed during the current study.
